# A20 deficiency sensitizes pancreatic beta cells to cytokine-induced apoptosis *in vitro* but does not influence type 1 diabetes development *in vivo*

**DOI:** 10.1038/cddis.2015.301

**Published:** 2015-10-15

**Authors:** L Catrysse, M Fukaya, M Sze, K Meyerovich, R Beyaert, A K Cardozo, G van Loo

**Affiliations:** 1 Inflammation Research Center, Unit of Molecular Signal Transduction in Inflammation, VIB, B-9052 Ghent, Belgium; 2 Department of Biomedical Molecular Biology, Ghent University, B-9052 Ghent, Belgium; 3 ULB Center for Diabetes Research, Université Libre de Bruxelles, B-1070 Brussels, Belgium

Dear Editor,

Type 1 diabetes mellitus (T1D) is an autoimmune disease characterized by the infiltration of inflammatory cells into the pancreatic islets of Langerhans, followed by the selective destruction of insulin-producing *β*-cells, resulting in hyperglycemia. One of the mechanisms causing *β*-cell death is the intra-islet release of inflammatory mediators such as interleukin-1*β* (IL-1*β*), tumor necrosis factor (TNF) and interferon-*γ* (IFN-*γ*) by activated immune cells.^1^ Hence, the transcription factor NF-*κ*B promotes pro-inflammatory and pro-apoptotic responses in *β*-cells on cytokine exposure. A transgenic mouse line in which NF-*κ*B activation is attenuated specifically in *β*-cells conferred nearly complete protection against multiple low dose streptozotocin (MLDSTZ)-induced T1D.^2^ Contrary, mice with constitutively active NF-*κ*B signaling in *β*-cells spontaneously develop full-blown immune-mediated diabetes.^3^

The ubiquitin-editing enzyme A20 is a critical negative regulator of NF-*κ*B signaling in response to multiple stimuli, including TNF and IL-1. Moreover, A20 can also act as a strong anti-apoptotic protein in specific cell types.^4^ A20 has been identified as the most highly upregulated anti-apoptotic protein in cytokine-stimulated primary islets and insulinoma cell lines.^5^ Consistent with this, overexpression of A20 in islets confers resistance to cytokine-mediated activation of NF-*κ*B, protecting them from apoptosis in the early post-transplantation period.^6^ Interestingly, not only have NF-*κ*B polymorphisms been identified in patients with T1D,^7^ also A20/*TNFAIP3* has been identified as a T1D susceptibility locus in humans.^8^ Together, these data suggest an important role for A20 in *β*-cell function and T1D. Therefore, we generated and characterized A20-deficient mice which lack expression of A20 specifically in *β*-cells (Supplementary Figure 1A).

We first confirmed the anti-apoptotic function of A20 in *β*-cells, as primary islets isolated from *β*-cell-specific A20 knockout (A20^*β*−KO^) mice were more susceptible to cytokine-induced cell death compared with wild-type islets (Supplementary Figure 1A). As A20 has a crucial role in *β*-cell survival *in vitro*, we next investigated whether A20^*β*-KO^ mice would be more susceptible to diabetes development when compared with wild-type littermates. A20^*β*-KO^ mice aged normally without any evidence of metabolic defects. Phenotypic analysis of A20^*β*-KO^ mice up to the age of 12 months revealed no pathological signs in the pancreas. A20^*β*-KO^ mice and control littermates were subjected to a model of T1D induced by MLDSTZ, however, both control and A20^*β*-KO^ mice developed a similar hyperglycemia, which was confirmed in a glucose tolerance test (ipGTT) performed 5 weeks after the first STZ injection (Supplementary Figure 1B). Next, we crossed A20^*β*-KO^ mice with C57BL6-Ins2^Akita^/J mice, which carry a mutation in the insulin *Ins2* gene that prevents normal folding and secretion and induces endoplasmic reticulum stress leading to *β*-cell death. Mice carrying the Ins2^Akita^ mutation become hyperglycemic very early in life, however, no differences could be observed in conditions of A20 deficiency in *β* cells. In agreement, ipGTT shows severe and similar defects in insulin secretion in both Ins2^Akita^ and A20^*β*−KO/Akita^ mice (Supplementary Figure 1C). Finally, A20^*β*-KO^ mice were backcrossed into a non-obese diabetic genetic background, and glucose levels were measured every week in order to follow diabetes development. Although only 40% of all mice developed diabetes, no differences could be detected between control and A20^*β*-KO^ mice (Supplementary Figure 1D). In conclusion, A20 deficiency in *β* cells does not affect *β*-cell apoptosis nor disease development *in vivo*.
