# Supplementary figures and images for: A20 deficiency sensitizes pancreatic beta cells to cytokine-induced apoptosis in vitro but does not influence type 1 diabetes development in vivo
Source: Cell Death Dis. 2015 Oct 15;6(10):e1918–. doi: 10.1038/cddis.2015.301 (PMC4632319; doi:10.1038/cddis.2015.301)

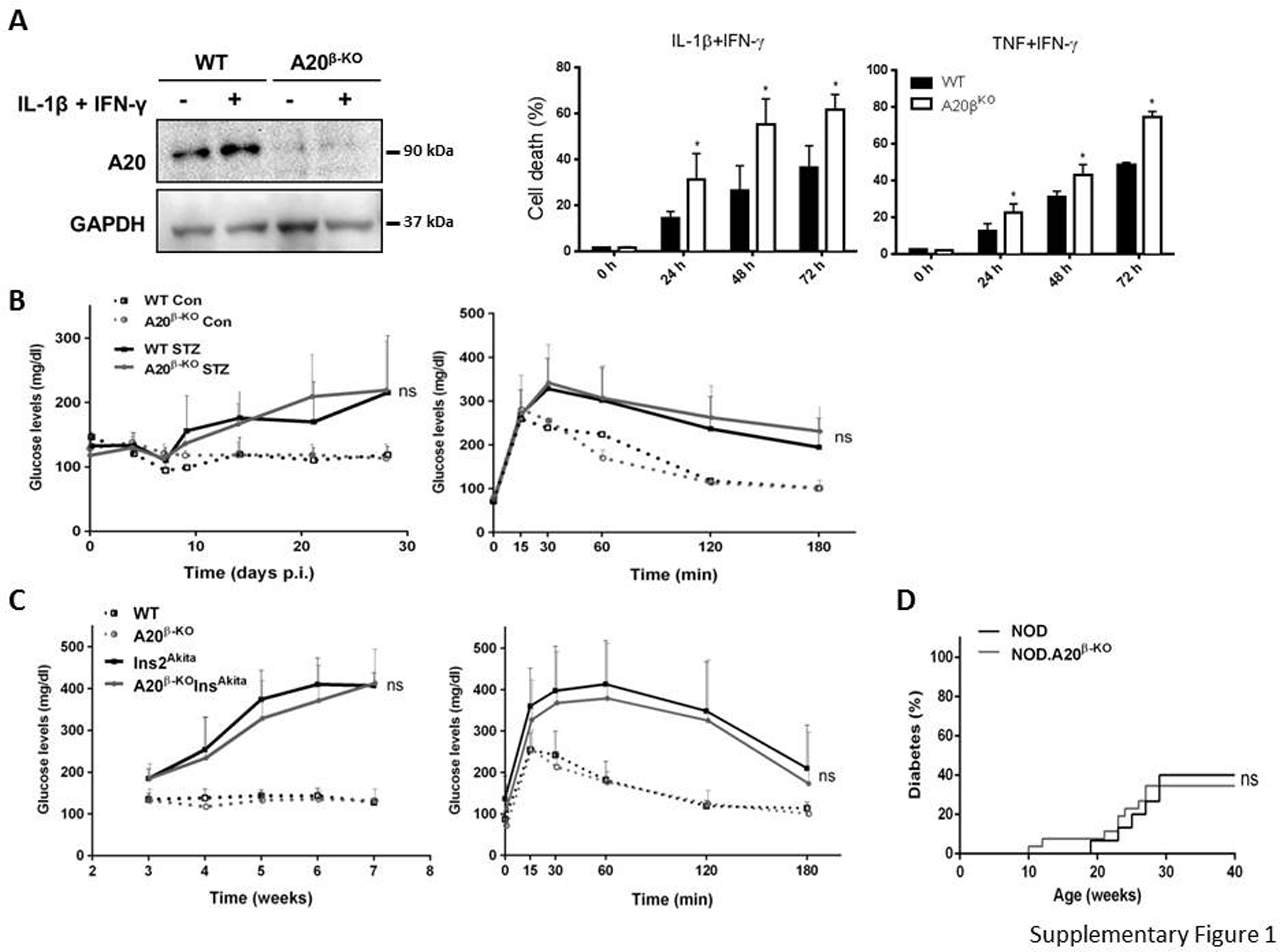

Supplement: Supplementary Figure 1 [file cddis2015301x1.tif]
