# Supplementary material for: A20 deficiency sensitizes pancreatic beta cells to cytokine-induced apoptosis in vitro but does not influence type 1 diabetes development in vivo
Source: Cell Death Dis. 2015 Oct 15;6(10):e1918–. doi: 10.1038/cddis.2015.301 (PMC4632319; doi:10.1038/cddis.2015.301)
Supplement: Supplementary Information [file cddis2015301x2.doc]

**SUPPLEMENTARY FIGURE 1.** ***A20 deficiency in β-cells sensitizes to β-cell apoptosis in vitro, but does not influence diabetes development.*** (A) Pancreatic islets from β-cell-specific A20 knockout (A20^β-KO^, KO) and wild type (WT) mice were isolated, cultured overnight, and either or not exposed to IL-1β (10 U/ml) or TNF (1000U/ml) and IFNγ (1000 U/ml) for the indicated times. Western blot analysis on islets from WT and A20^β-KO^ mice exposed to IL-1β+IFN-γ for 24h (left). Percentage of cell death evaluated by Hoechst-Propidium iodide staining (right). Data are mean ± S.E.M. of 3-4 independent experiments. * *p* < 0.05 vs WT. (B) A20^β-KO^ and WT littermates were injected with 42.5 mg/kg of streptozotocin (STZ, n=11-13) or control buffer (Con, n=2-4) for 5 subsequent days. Glucose levels were measured at day 0, 4, 7, 9, 14, 21 and 28 days post injection in non-starved conditions (left). Intra-peritoneal Glucose Tolerance Test (ipGTT) was performed 5 weeks post injection (n= 7-8, Con: n= 2) (right). (C) A20^β-KO^, Ins2^Akita^, A20^β-KO^ Ins2^Akita^ and WT littermates (n= 8-9) were grown, and non-starved glucose levels were measured weekly, starting from 3 weeks of age (left). Intra-peritoneal Glucose tolerance test (ipGTT) was performed at 6 weeks (right). (D) A20^β-KO^ (n=21) and control littermate mice (n=17) were backcrossed into a NOD background and diabetes development was analysed based on blood glucose levels. Data are presented as mean ± SD and 2-way ANOVA was used as a statistical test.
